# Contraceptive uses among married women in Bangladesh: a systematic review and meta-analyses

**DOI:** 10.1186/s41043-024-00502-w

**Published:** 2024-01-17

**Authors:** Sorif Hossain, Tahmina Akter, Md Mohsin, Md. Momin Islam, Promit Barua Chowdhury, Md Mohsan Khudri

**Affiliations:** 1 Department of Statistics, Noakhali Science and Technology University, Noakhali, Bangladesh; 2 Interdisciplinary Health Sciences, The University of Texas at El Paso, El Paso, USA; 3 Department of Meteorology, University of Dhaka, Dhaka, 1000 Bangladesh; 4 Institute of Statistical Research and Training, Dhaka, Bangladesh; 5 Department of Economics, Fogelman College of Business and Economics, The University of Memphis, Memphis, USA

**Keywords:** Contraceptive use, Women, Bangladesh, Meta-analyses, Trends

## Abstract

**Background:**

Although Bangladesh's economy has shown significant improvement over the past two decades, the high population growth rate has hindered development efforts. This study aimed to review the prevalence of different contraceptive methods used among women of reproductive age in Bangladesh, which could help control the population growth rate.

**Methods:**

We conducted an extensive literature review and meta-analysis of 82 articles, identifying 20 articles for analysis. The analyses included heterogeneity and publication bias in published papers.

**Results:**

The pooled prevalence of various contraceptive methods was as follows: condom use, 7.13%; Female Sterilization, 8.09%; injectables, 12.76%; intrauterine devices (IUDs), 3.76%; male sterilization, 2.34%; periodic abstinence, 6.71%; pills, 33.21%; and withdrawal, 3.27%. Modern contraceptive methods accounted for 62.91% of usage, while traditional methods constituted 8.79%. On average, only 1.95% of women opted for the implant method. The overall prevalence of contraceptive method usage was 59.48%, with 60.59% in urban areas and 54.54% in rural areas. We found statistically significant heterogeneity for all contraceptive methods used by women in Bangladesh. The funnel plot and Egger’s test showed no publication bias for any of the contraceptive methods, except condoms (*Z* = 2.34, *P* = 0.0194). The contraceptive methods used by women in rural areas also showed publication bias (*Z* = −3.04, *P* = 0.0024).

**Conclusion:**

A renewed commitment from government bodies and independent organizations is needed to implement and monitor family planning strategies to ensure adherence to and provision of the most appropriate contraceptive method for couples.

**Supplementary Information:**

The online version contains supplementary material available at 10.1186/s41043-024-00502-w.

## Introduction

Contraception indicates to prevent pregnancy as a consequence of sexual intercourse [6, 8]. Contraception can generally be described as the intentional prevention of conception or impregnation during sexual activity, through man-made means such as using various devices, agents, drugs, sexual practices, or surgical procedures. The main purpose of these methods is to prevent the sperm from reaching the ovum by using condoms, diaphragms, inhibiting ovulation, and so on [6, 8]. Different types of contraception that an individual can use to prevent pregnancies are condoms, oral contraceptive pills, intrauterine devices (IUD), contraceptive implants, contraceptive injections, emergency contraception pills (The 'Morning After' Pill), contraceptive rings, diaphragm, sterilization, etc. [22]. Contraception is linked to family planning. Contraception and voluntary sterilization can manage the number of children and their birth intervals, minimizing mother and newborn mortality. Family planning accelerates progress across all five SDG themes—People, Planet, Prosperity, Peace, and Partnership—making it crucial to sustainable development [39]. Out of the 1.9 billion women of reproductive age (15–49 years) worldwide in 2019, 1.1 billion need family planning, either as current users (842 million using modern methods and 80 million using traditional methods) or as unmet needs (190 million wanting to avoid pregnancy without contraception) [44]. In developing countries, contraceptive use prevented 218 million unintended pregnancies in 2012, averting 55 million unplanned births, 138 million abortions (of which 40 million were unsafe), 25 million miscarriages, and 118,000 maternal deaths [45, 47]. In 2017, it was found that about 63% of married women of reproductive age or their partners used either modern or traditional methods of contraception [47].

Regional and national differences masked global progress. Around 225 million low- and middle-income countries (LMIC) women do not use modern contraception to avoid pregnancy. Approximately 40 million abortions take place each year, with half of them being unsafe and illegal, along with 30 million unplanned pregnancies annually. Girls and women make up about half of the 499 million new sexually transmitted disease (STD) cases (excluding HIV) reported annually [34, 43]**.** In South Asia, the prevalence estimates of modern contraceptive (%) methods used among women of reproductive age (15–49 years) were: in Afghanistan (18.2%), Bangladesh (52.8%), Bhutan (42.7%), India (42.6%), Nepal (40.5%), Pakistan (23.6%), Maldives (31.9%), and Sri Lanka (46.3%), respectively [44].

In Bangladesh, the proportion of married women who reported using a family planning method at the time of the study is defined as current contraception use. According to the 2017–2018 Bangladesh Demographic Health Survey (BDHS) [29], 62% of currently married women aged 15–49 used contraception. Modern techniques are used by 52% of married women today. The pill (25%) is the most popular method, followed by injectables (11%). A long-acting or permanent method, such as female or male sterilization, implant, or IUD, is used by 9% of married women. Traditional methods are used by 10% of women, while rhythm methods are used by the majority (7%) of women (periodic abstinence) [29]. Fertility rates and high population growth affect human development and adversely impact the health and lives of women [46]. The fertility rate for Bangladesh in 2014 was 2.18 births per woman and the current fertility rate for Bangladesh in 2023 is 1.93 births per woman which means a 11.47% decline from 2014 [25]. Although the proportion decreased over the year, with a growth rate of 1.37 per annum, Bangladesh is one of the most densely populated countries in the world [1]. Despite improving Bangladesh's economy, the population growth rate has frustrated all development efforts. The government of Bangladesh has stated that population growth is the number one problem for the country; however, attempts to control this rapid growth rate have not been very successful. Several studies on contraceptives have found that the main reason behind the high population growth rate is the low use of contraceptives, especially in the rural areas where the overwhelming majority of the country's population lives [3, 15].

Although contraceptive use has a significant contribution to reducing population growth and achieving SDGs, only some of the studies in Bangladesh explored this [8, 9]. Moreover, in recent years, there has been a notable decline in studies and discussions on contraceptive use and family planning in Bangladesh. Through a systematic review and meta-analysis of available literature, this study aims to give a full picture of the patterns and prevalence of contraceptive use among married women in Bangladesh.

## Methods

Preferred Reporting Items for Systematic Reviews and Meta-Analyses (PRISMA) were followed for conducting this meta-analysis and systematic reviews [24].

### Search strategy and selection criteria

This systematic review comprehensively examines published research papers on the prevalence of various contraceptive practices, including modern, traditional, and folkloric methods, female sterilization, male sterilization, injectables, intrauterine contraceptive devices (IUD), pills, condoms, periodic abstinence, and implants. The search, initiated on May 1, 2022, focused primarily on the MEDLINE database (PubMed), Medical Literature Analysis and Retrieval System Online (MEDLINE), and Google Scholar.

To identify relevant studies, a combination of keywords such as "contraceptive uses," "women," "married women," "contraception," "contraceptive methods," "birth control," and "Bangladesh" was systematically employed. An example of the keyword combination used is as follows:

- (((((Prevalence [Title]) AND (Contraceptive uses [Title])) OR (Contraception [Title])) OR (Birth control [Title]) OR (Contraceptive methods [Title]) OR (Bangladesh [Title])))

Google Scholar and EndNote reference management software (Version X8.1) were utilized for the search, employing various combinations of keywords related to contraceptive use among women in Bangladesh. The inclusion criteria for studies encompassed the period from 1990 to 2022, and only studies published in English were considered.

### Inclusion and exclusion criteria

To ensure the thoroughness and relevance of our study, we applied precise inclusion and exclusion criteria. Studies were included only if they covered contraceptive uses, encompassing modern, traditional, and folkloric methods, female sterilization, male sterilization, injectables, intrauterine contraceptive devices (IUD), pills, condoms, periodic abstinence, and implants. Exclusion criteria were applied if the full text was unavailable, if the study was unrelated to the subject if it lacked essential data (such as research design, study site, or sample size), or if it failed to provide at least one of the following details regarding contraceptive uses: (1) The prevalence of contraceptive uses, (2) The prevalence of urban and rural contraceptive use rates, and (3) The prevalence of modern, traditional, and folkloric methods, female sterilization, male sterilization, injectables, intrauterine contraceptive devices (IUD), pills, condoms, periodic abstinence, and implants.

In addition to these criteria, various non-research and non-peer-reviewed materials were excluded to uphold the study's integrity. This exclusion encompassed review papers, letters to the editor, brief reports, editorials, comments, correspondence, local reports, master's and doctoral theses, conference abstracts, and presentations. These exclusions were implemented to maintain the rigour and reliability of the analysis.

### Study selection procedure

**Fig. 1:**
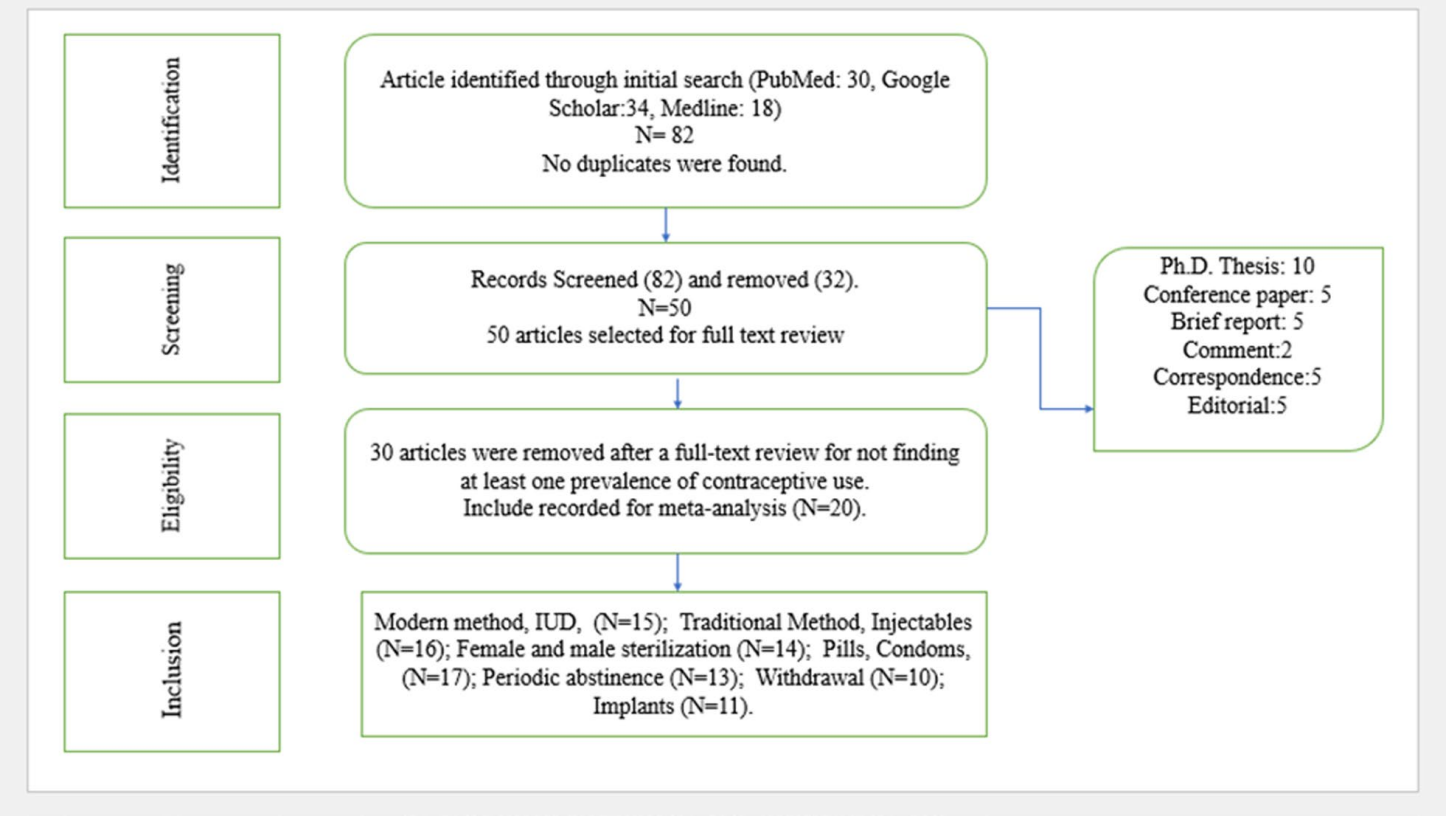
Study selection procedure

This systematic review and meta-analysis adhered to the Preferred Reporting Items for Systematic Reviews and Meta-Analyses (PRISMA) guidelines, as illustrated in Fig. 1, to guide the collection and assessment of publications. The initial search yielded 82 items, with no duplicate records identified. After the initial evaluation, 32 studies were excluded. Subsequently, during the screening phase, 50 articles were selected, and in the eligibility phase, 30 studies were disqualified due to missing information on at least one of the following: prevalence of contraceptive methods (pills, condoms, IUDs, implants, etc.), research duration, sampling technique, study location, or study period.

The final analysis included 20 papers that satisfied all necessary criteria, forming the basis for our comprehensive analysis. The selected studies provided data on various parameters, including the first author's name, region, sampling design, sampling method, study duration, sample size, age groups, prevalence of contraceptive uses (modern, traditional, folkloric, female and male sterilizations, injectables, IUDs, pills, condoms, periodic abstinence, withdrawal, and vasectomy), contraception uses in urban and rural areas, study quality, title, and paper link.

### Screening and extraction

Initially, one author screened the titles and abstracts to eliminate duplicate entries and irrelevant themes (SH). Two independent authors (TA and SH) checked accuracy and inconsistencies. However, no inconsistencies were found by them. Then two independent reviewers (SH and PBC) did the full-text review and confusion, or inconsistency was resolved by consulting with the reviewer TA. Data were extracted from different sources in the Excel file (standard data extraction form). Finally, this data was also cross-checked by the reviewer SH.

### Statistical analysis

*Q*-test and *I*^2^ statistics with a 5% level of significance were used to assess the between-study heterogeneity [10, 26]. To estimate the pooled prevalence of various contraceptive methods, including modern methods, pills, condoms, and more, we utilized a random-effects model with 95% confidence intervals and the relative weight assigned to each study. We presented the results of our meta-analyses in the form of tables and forest plots [40]. The probable publication bias was found using the funnel plot and Egger's test [26, 41]. In our subgroup analysis, we examined the prevalence of contraceptive methods across various strata, considering study duration and sampling methods. Specifically, we examined trends in the prevalence of contraceptive use, including pills, condoms, IUDs, implants, and more, by categorizing all studies into different groups based on their respective study durations. Additionally, we explored the patterns of contraceptive usage in both rural and urban areas. All statistical analyses were conducted using the statistical software package STATA (Version 16.0).

## Results

### Study characteristics

The features of the chosen studies are described in Table 1. Out of the 20 studies, 15 were conducted throughout Bangladesh [2, 5, 7, 11, 12, 16–18, 23, 27, 29–33, 42], with the remaining five focusing on selected areas, including Gazipur, Matlab, Sylhet, Rajshahi, and Narshingdi [13, 19–21, 35]. This meta-analysis included studies conducted from 1990 to 2022. A total of 193,348 samples were covered under all eligible studies. Various sampling techniques were used in the chosen literary works including cross-sectional, two-stage sampling, stratified sampling, simple random sampling, purposive sampling, and systematic sampling.

**Table 1:** Features of the studies included in the meta-analyses

Author name	Region	Sampling design	Study duration	Sample size	Age groups	Prevalence of modern method (95% CI)	Traditional contraceptive methods (95% CI)	Folkloric (%)	Female sterilization (95% ci)	Male sterilization (95% ci)	Injectables (95% CI)	IUD (95% CI)
Islam et al. 11	Bangladesh	Cross Sectional	2011	16,616	15–49	84.77 (84.23, 85.32)	14.61 (14.07,15.15)	0.6	7.97 (7.56, 8.38)	1.93 (1.72, 2.14)	18.27 (17.86, 18.86)	1.24 (1.07, 1.41)
Haq et al. 5	Bangladesh	Time series	1994– 2014	38,648	15–49							
Khan and Jerifa [19]	Gazipur, sharper, Bangladesh	Cross sectional data	2012	265	15–49		1.2 (−.11, 2.51)		7.3 (4.17, 10.43)		2.4 (0.56, 4.24)	
Islam [12]	Bangladesh	Cross sectional data	2011	3507	15–49							
Mostafa Kamal [27]	Bangladesh	Cross sectional data	2007	1424	15–49	38 (35.48, 40.52)	4 (2.98, 5.02)					
Kamruzzama and Hakim 16	Bangladesh	Cross sectional	Jan 2014–Dec2014	350	19–49						10 (6.86, 13.14)	3.43 (1.52, 5.34)
Koenig et al. [21]	Matlab, Chandpur, Bangladesh	Cross sectional	1990	4238	19–49	94.2 (93.5, 95)	5.7 (5, 6.40)		14.8 (13.73, 15.87)		49.8 (48.29, 51.31)	6.2 (5.47, 6.93)
Laskar et al. [23]	Bangladesh	Cross sectional	2006	8748	19–49				11.83 (11.15, 12.51)	10.5 (9.86, 11.14)	13.32 (12.61, 14.03)	24.7 (23.80, 25.60)
Hoq [7]	Bangladesh	Cross sectional	2018	16,858	19–49	54 (53.25, 54.75)	8.4 (7.98, 8.82)		4.6 (4.28,4.92)	1.2 (1.04, 1.36)	12.4 (11.90, 12.90)	0.6 (0.48, 0.72)
Callahan and Becker [2]	Bangladesh	Longitudinal	2006–2009	3080	13–49				8.1 (7.14, 9.06)	0.2 (0.04, 0.36)	20.8 (19.37, 22.23)	2.6 (2.04, 3.16)
Islam et al. [14]	Bangladesh	Longitudinal and surveillance system	1998	13,515	15–49	34.7 (33.9, 35.5)	5.3 (4.92, 5.68)				7.5 (7.06, 7.94)	2.5 (2.24, 2.76)
Khan and Shaw [17]	Bangladesh	Cross sectional	2009	11,440	15–49	79.175 (78.43, 79.92)	19.78 (19.05, 20.51)	1.025				
Kibria et al. [20]	Shyllet division, Bangladesh	cross sectional	2017	1147	15–49	85.5 (83.46,87.54)	14.4 (12.37, 16.43)		6.7 (5.25, 8.15)	1.2 (0.57, 1.83)	6.5 (5.07, 7.93)	0.2 (−0.06, 0.46)
Khan and Islam 18	Bangladesh	Cross sectional	2022	5574	15–49	88.6 (87.77, 89.43)	11.39 (10.56, 12.22)		12.57 (11.07, 13.44)			
Rahman 35	Bangladesh	Interviewing	2010	1000	15–19	52.7 (49.61, 55.79)	4.7 (3.39, 6.01)				0.7 (0.18, 1.22)	0.7 (0.18, 1.22)
Islam [13]	Narsingdi, Bangladesh	face to face Interview	2012	430	15–49	50.9 (46.17, 55.63)	10.2 (7.34, 13.06)		6.4 (4.09, 8.71)	0.93 (0.02, 1.84)	5.58 (3.41, 7.75)	3.48 (1.75, 5.21)
Streatfield et al. [42]	Bangladesh	Follow up survey	2015	26,072	19–49	69 (68.44, 69.56)	5 (4.74, 5.26)		10 (9.64, 10.36)	5 (4.74, 5.26)	15 (14.57, 15.43)	5 (4.74, 5.26)

Author name	Pills (95% CI)	Condoms (95% CI)	Periodic abstinence 95 (% CI)	Withdrawal (95% CI)	Implants (95% CI)	Vasectomy	Others	Contraceptive uses (95% CI)	Urban (95% CI)	Rural (95% CI)	Study Quality (15–30)
Islam et al. 11	43.72 (42.97, 44.47)	9.8 (9.35, 10.25)	11.57 (11.08, 12.06)	3.037 (2.78, 3.30)	1.82 (1.62, 2.02)		0.6	61.62 (60.88, 62.36)	64.5 (63.77, 65.23)	60.25 (59.51, 60.99)	28
Haq et al. 5									59.28 (58.79, 59.77)	51.84 (51.34, 52.34)	26
Khan et al. [19]	69 (63.43, 74.57)	10.9 (7.15, 14.65)			2.4 (0.56, 4.24)		1.9	62.3 (56.46, 68.14)			20
Islam [12]									71.8 (70.31, 73.29)	66.9 (65.34, 68.46)	26
Mostafa Kamal [27]											22
Kamruzzama and Hakim 16	20 (15.81, 24.19)	4.29 (2.17, 6.41)	2 (0.53, 3.47)		3.14 (1.31, 4.97)			45.71 (40.49, 50.93)	58 (52.83, 63.17)	30 (25.20, 34.80)	25
Koenig et al. [21]	21.1 (19.87, 22.33)	1.4 (1.05, 1.75)				0.7		57.1 (55.61, 58.59)			26
Laskar et al. [23]	41.86 (40.83, 42.89)	9.03 (8.43, 9.63)	10.41 (9.77, 11.05)	7.7 (7.14, 8.26)			0.14	59.61 (58.58, 60.64)			27
Hoq [7]	27 (26.33, 27.67)	6.4 (6.03, 6.77)	6.2 (5.84, 6.56)	1.9 (1.69, 2.11)	1.7 (1.50, 1.90)		0.3	62.4 (61.67, 63.13)			22
Callahan and Becker [2]	47.8 (46.04, 49.56)	6.7 (5.82, 7.58)	9.6 (8.56, 10.64)	1.6 (1.16, 2.04)	1.1 (0.73, 1.47)			72.8 (71.23, 74.37)			20
Islam et al. [14]	14.3 (13.71, 14.89)	2.7 (2.43, 2.97)						39.9 (39.07,40.73)			24
Khan and Shaw [17]											27
Kibria et al. [20]	21.36 (18.99, 23.73)	3.9 (2.78, 5.02)	5.6 (4.27, 6.93)	1.2 (0.57, 1.83)				58.5 (55.65, 61.35)	45 (42.12, 47.88)		28
Khan and Islam 18	64.8 (63.55, 66.05)	11.5 (10.66, 12.34)	11.3 (10.47, 12.13)								23
Rahman 35	28 (25.22, 30.78)	22.6 (20.01, 25.19)	2.7 (1.70, 3.70)		2 (1.13, 2.87)		56.7	60 (56.96, 63.04)	55 (51.92, 58.08)		24
Islam [13]	26.51 (22.34, 30.68)	7.2 (4.76, 9.64)	3.2 (1.54, 4.86)	6.27 (3.98, 8.56)	0.46 (−0.18, 1.10)		61.1				26
Streatfield et al. [42]	25 (24.47, 25.53)	4 (3.76, 4.24)			5 (4.74, 5.26)			74 (73.47)			20

### Statistical heterogeneity and publication bias

**Fig. 2:**
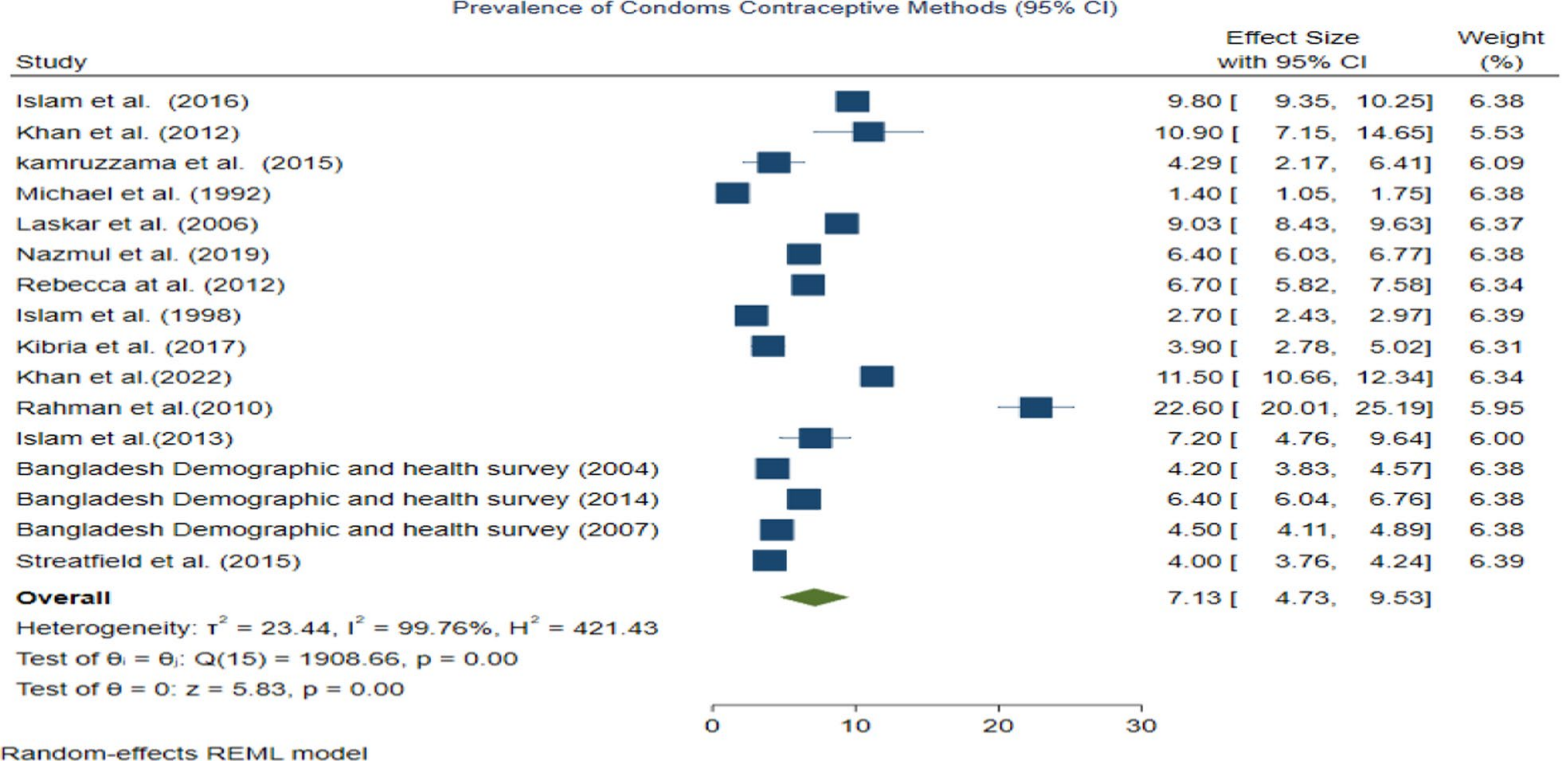
Forest plot showing the results of the pooled prevalence of Condom methods of contraceptive use among women in Bangladesh

**Fig. 3:**
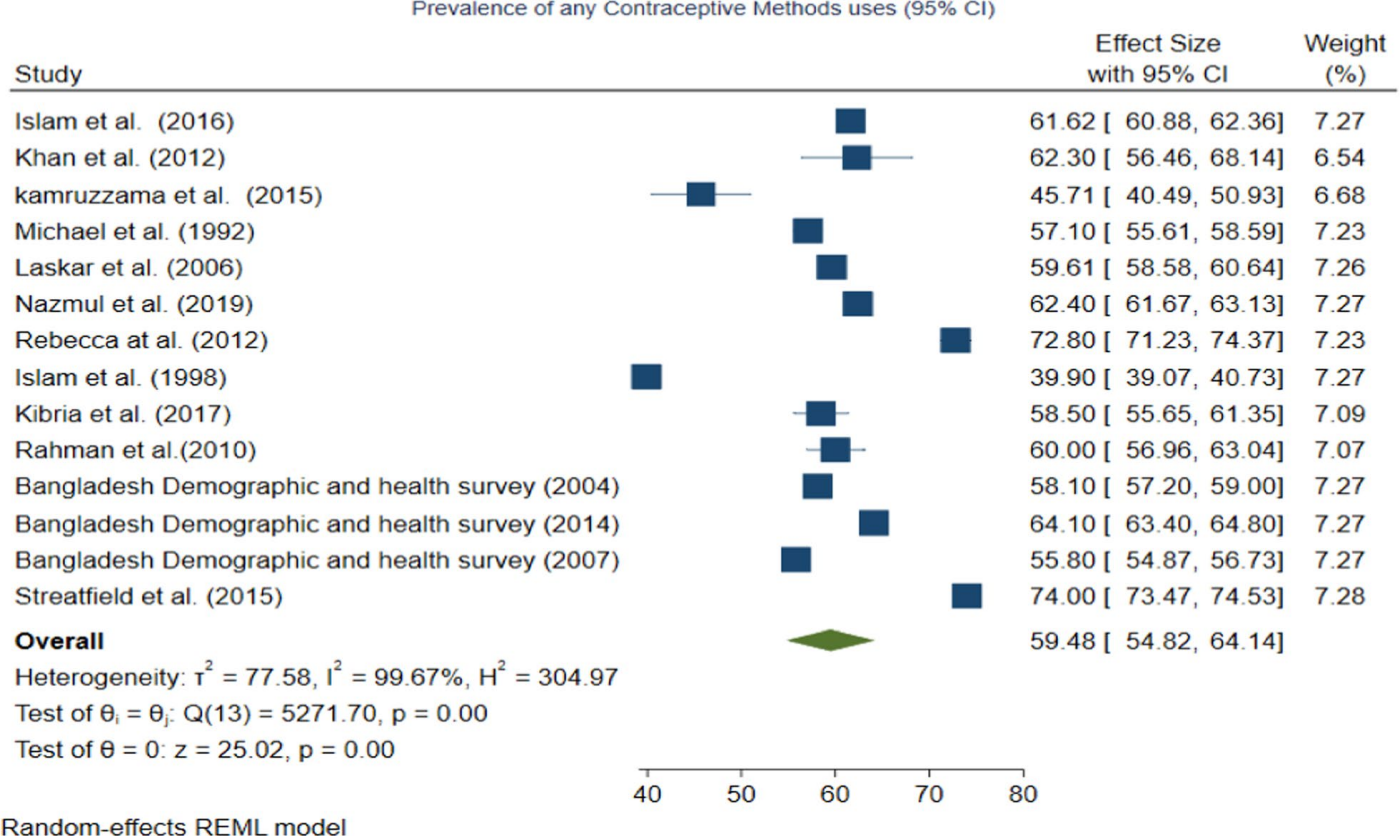
Forest plot showing the results of the pooled prevalence of any methods of contraceptive uses among women in Bangladesh

**Fig. 4:**
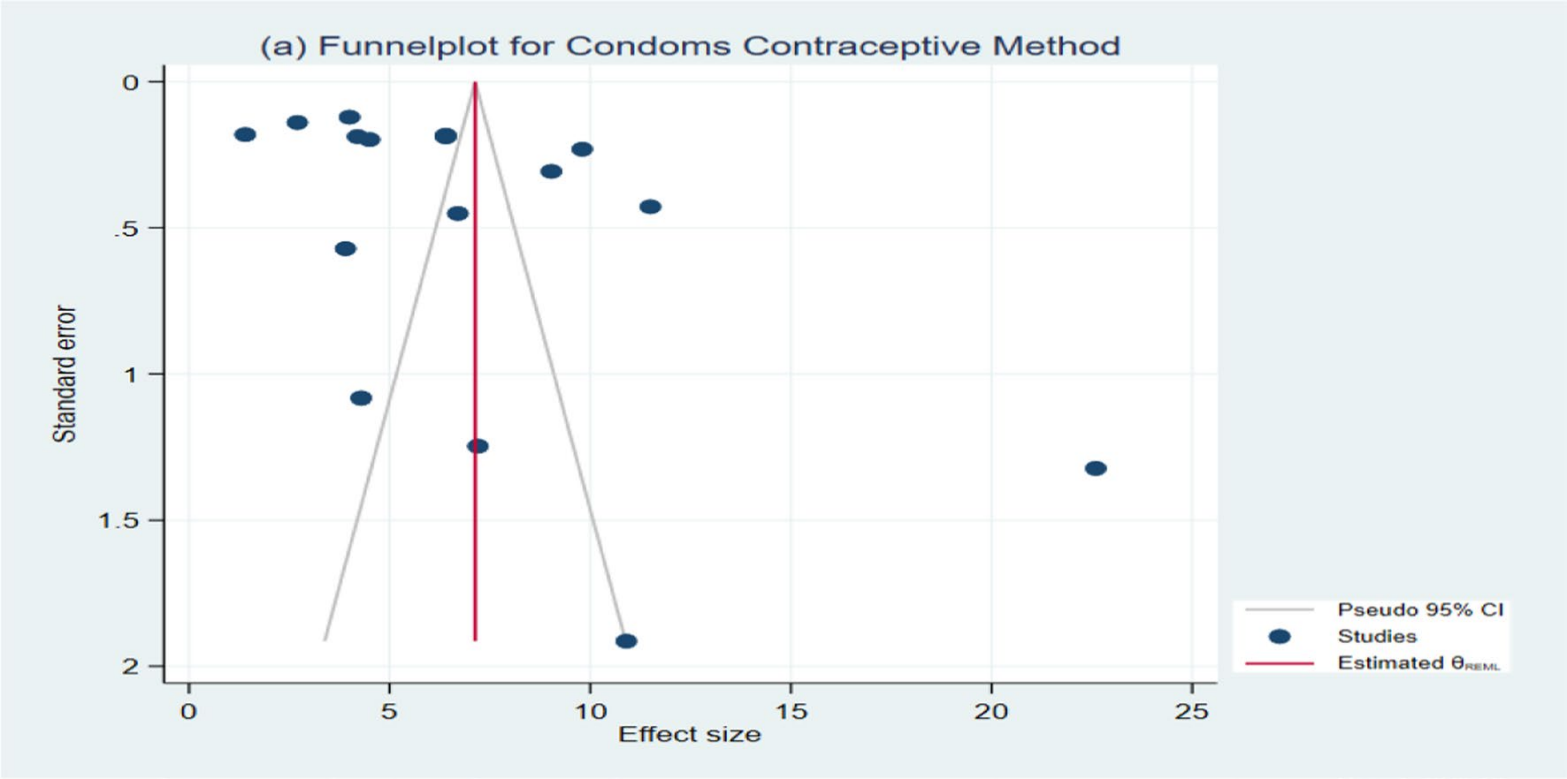
Funnel plot of result of the prevalence of Condoms methods of contraceptive uses among women in Bangladesh

**Fig. 5:**
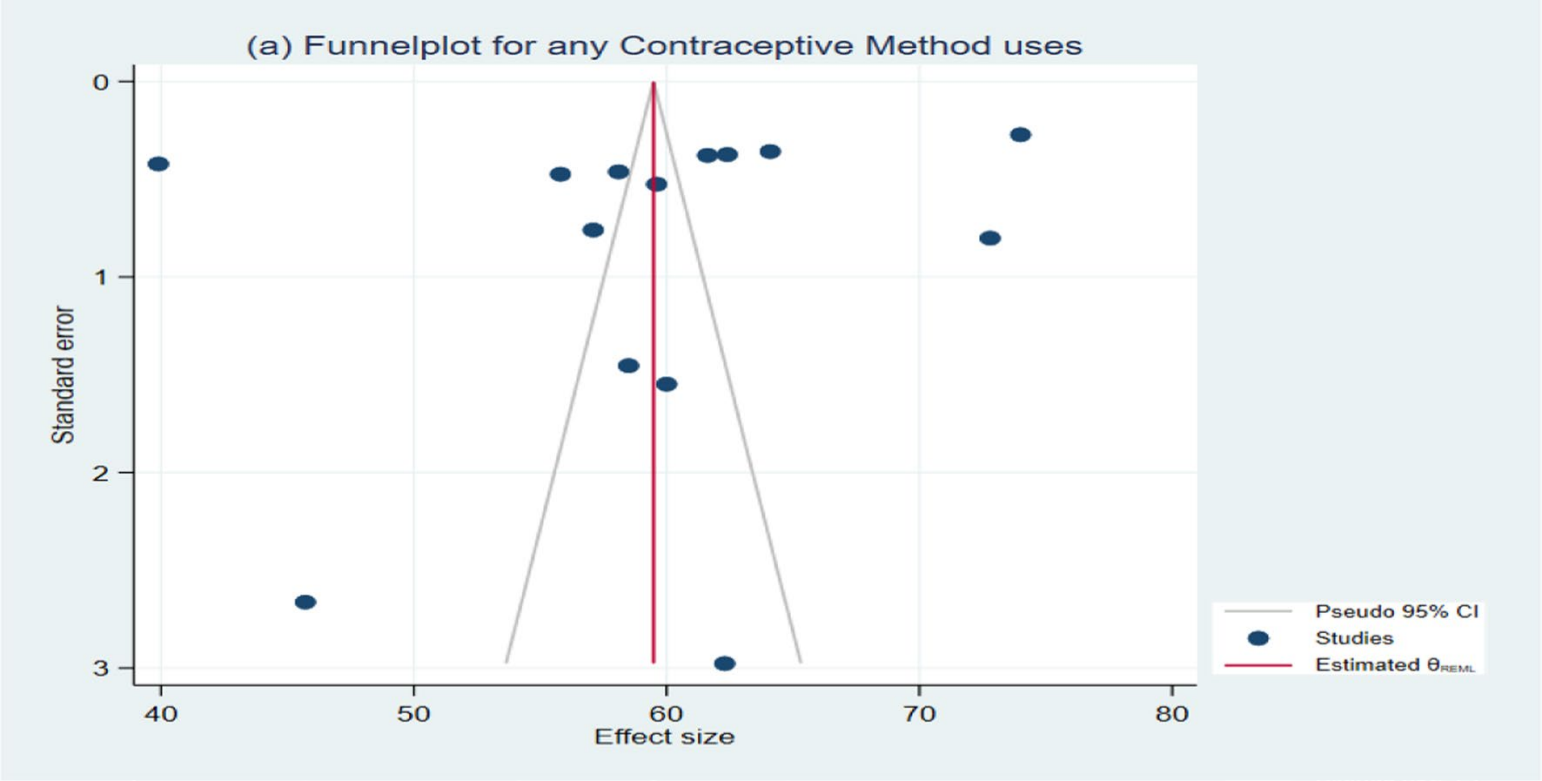
Funnel plot of result of the prevalence of any methods of contraceptive uses among women in Bangladesh

To assess heterogeneity among studies, we employed statistical methods, including the *Q*-test and *I*^2^ (%) statistic, along with their respective *p* values. Our analysis revealed significant heterogeneity across all contraceptive methods used by women in Bangladesh (e.g., Condoms: *I*^2^ = 99.76%, *Q* (15) = 1908.66, *P* = 0.00; Pills: *I*^2^ = 99.91%, *Q* (15) = 8744.61, *P* = 0.00, and so forth), as depicted in Figs. 2 and 3 (Additional file 1: Figure S7-S18). Additionally, a funnel plot and Egger’s test were employed to evaluate publication bias. The findings indicated no publication bias for most contraceptive methods, except for condoms (*Z* = 2.34, *P* = 0.0194), as illustrated in Figs. 4 and 5 (Additional file 1: Figures S19-S30). Notably, contraceptive methods used by women in rural areas also exhibited publication bias (*Z* = −3.04, *P* = 0.0024). Detailed results of Egger’s test, assessing publication bias, can be found in the Additional file 1.

Moreover in our study, we also detected some outliers. For example, a forest plot for condom contraceptive methods showed Rahman [35] reported a high prevalence of condom contraceptive use as compared to other included studies, which was one of the causes of examined heterogeneity among studies. Similarly, studies namely Streatfield et al. [42], Koenig et al. [21], Laskar et al. [23], Kamruzzman and Hakim 16, and Khan and Shaw [17] showed a higher prevalence of Implants, Injectables, IUDs and male sterilization and withdrawal, in rural areas, traditional contraceptive methods than other included studies. These outliers are also one of the causes of heterogeneity.

### Prevalence

The pooled prevalence of any contraceptive method used estimated from the 20 studies was 59.48% (CI 54.82, 64.14, *I*^2^: 99.67%) (Fig. 3). The pooled prevalence of contraceptive uses in urban areas was 60.59% (CI 55.66, 65.53, *I*^2^: 99.58%), while for rural women it was 54.54% (CI 45.97, 63.11, *I*^2^: 99.86%) (Additional file 1: Figure S15-S16)). The prevalence of modern contraceptive methods used was 62.91% (CI 52.39, 73.42, *I*^2^: 99.95%) and the prevalence of traditional methods used was 8.79% (CI 6.32, 11.27, *I*^2^: 99.65%) (Additional file 1: Figures S12 and S17). The pooled prevalence of condoms used was 7.13% (CI 4.73, 9.53; *I*^2^: 99.76%) estimated from 15 studies. Female Sterilization methods were used by 8.09% overall (CI 6.27, 9.92, *I*^2^: 99.36%) assessed from 12 studies, while male sterilization was used by 2.34% (CI 0.39, 4.29, *I*^2^: 99.89%). Only 1.95% of women used the implant method on average (CI 1.10, 2.81; *I*^2^: 98.87%). Among other methods, the pooled prevalence of injectables; IUD; periodic abstinence; pills; and withdrawal were 12.76% (CI 6.88, 18.64, *I*^2^: 99.92%); 3.76% (CI 0.46, 7.06, *I*^2^: 99.97%); 6.71% (CI 4.84, 8.59, *I*^2^: 99.19%); 33.21% (CI 25.49, 40.94, *I*^2^: 99.91%); and 3.27% (CI 1.85, 4.68, *I*^2^: 99.40%) (Figs. 2 and 3, Additional file 1: Figures S7-S18).

### Trends in the prevalence of contraceptive use among women in urban and rural areas

**Fig. 6:**
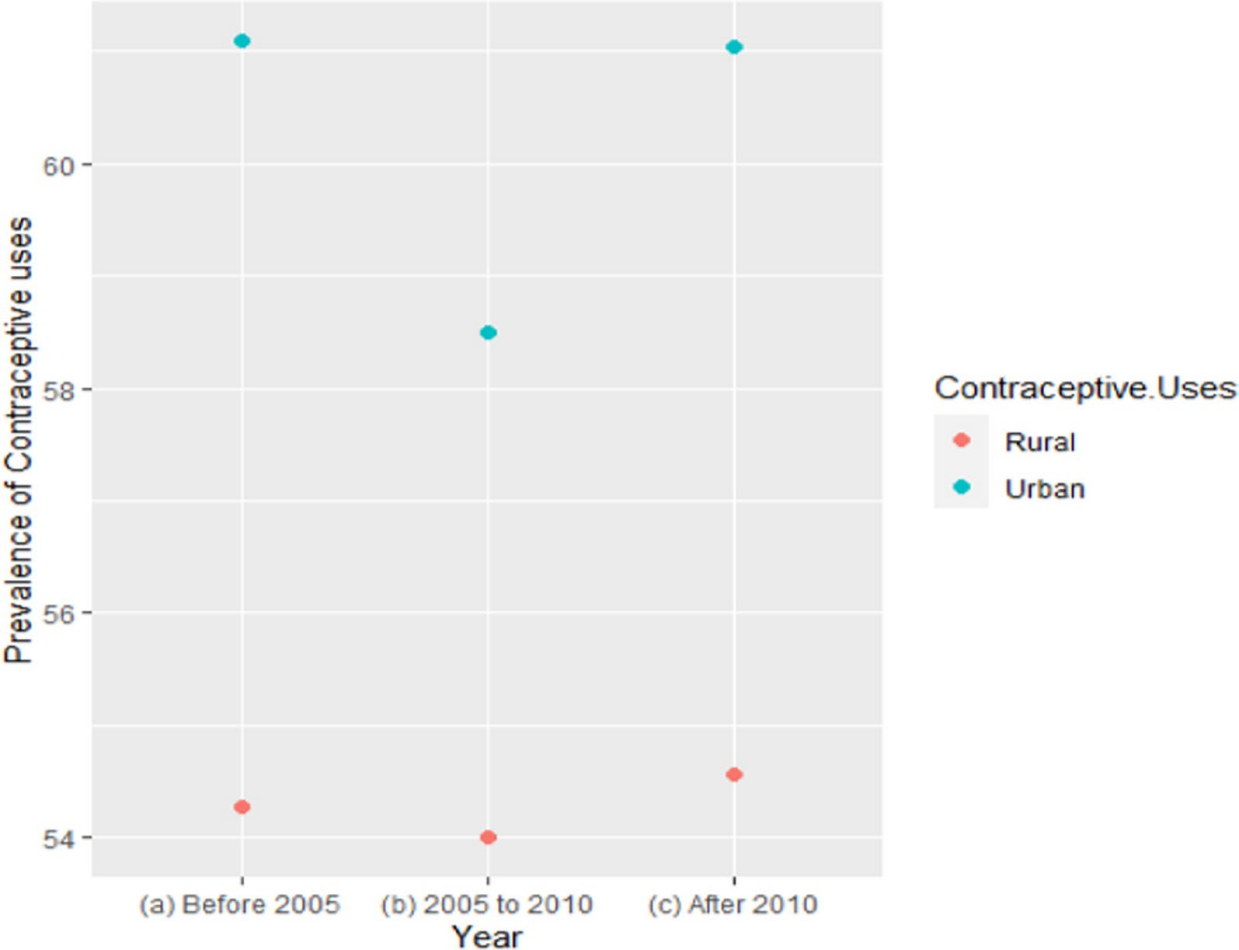
Trends in the prevalence of contraceptive uses among urban and rural area women (1990–2022)

The usage of contraceptives among rural women was lower than that of urban women in all three time periods (as shown in Fig. 6). Specifically, in each of the three time periods, the usage of contraceptives among rural women was below 55%, while among urban women, it was above 60% (reaching just under 59% from 2005 to 2010). Although contraceptive use in rural areas decreased before 2005 and from 2005 to 2010, it increased after 2010 in Bangladesh.

## Discussion

This systematic review and meta-analysis found that about 60% of reproductive-age women in Bangladesh use contraceptive methods. Substantial disparities in the prevalence of contraceptive usage were found between rural and urban reproductive age married women. Slow progress was seen in elevating the prevalence of contraceptive usage in recent years in Bangladesh.

Although the prevalence of contraceptive use in Bangladesh is lower than global estimates [28], it is higher than in most other Southeast Asian countries [4]. However, there is a substantial amount of contraceptive method failure in Bangladesh. In 2011, the rate of contraceptive method failure in Bangladesh was 22.8%, which increased to 27.3% in 2017/18. Contraceptive method failures cause unintended pregnancies, and such pregnancies give adverse outcomes (abortions, pregnancy complications, maternal and early childhood morbidity, mortality, etc.) [18]. Unplanned pregnancies happen when access to effective contraception is generally limited or when contraceptive methods are not utilized properly or consistently. As a result of inadequate contraceptive use, method failure, or non-use of contraception, it has been estimated that over 40% of unintended pregnancies happen worldwide each year [28]. Hence, in addition to increasing the contraceptive use rate, efforts should also be made to ensure the proper use of contraceptive methods.

The urban–rural disparities in contraceptive use prevalence and methods have always been a matter of concern for Bangladesh and other developing countries. This study also found a considerable gap in the usage of contraceptive methods between urban women (about 61% after 2010) and rural women (just over 54% after 2010) in Bangladesh. These estimates are consistent with previous studies conducted in Bangladesh, Southeast Asia, and other developing countries [20, 36, 37]. There might also be stronger disparities in contraceptive method failures among rural women due to a lower socio-economic status, knowledge gap, limited exposure to mass media and internet and healthcare facilities, etc. This systematic review found that about two-thirds of women used modern contraceptive methods while less than one-tenth used traditional methods. The prevalence of modern contraceptive method usage is comparatively higher among Bangladeshi women compared to their Southeast Asian counterparts (47%) [28]. Pills were taken by one-third of reproductive-age women among this study's participants. In addition, female sterilization and male sterilization were adopted by 8.09%, and 2.34%, respectively. From these findings, it is clear that in Bangladesh mainly female partners are responsible for adopting contraceptive methods. Condoms were used only by 7.13% of participants in this study. The use of condoms has been identified as the sole potential defense against sexually transmitted diseases (STDs). Globally, developing countries have a greater rate of STD prevalence among women than developed nations [4]. Such a poor prevalence of condom use in Bangladesh poses a significant threat to STDs including HIV and AIDS. Some societal factors, including gender disparity, ethnic and religious issues, lack of partner discussion about condom usage, and the stigma associated with the condom, were linked to not using a condom during sexual intercourse [38]. Efforts should be taken to elevate the use of condoms as a safe short-term contraceptive method and to tackle the threats of STDs.

In 2015, the 17 Sustainable Development Goals (SDGs) were adopted that chart a courageous course for a future that values fairness and inclusion, health, including sexual and reproductive health and reproductive rights, education, and more equality. The international community set a benchmark for modern contraceptive use of 75% by 2030 [34]. To attain the objective, between 2014 and 2030, demands served by modern means of contraception would need to rise by 2.2 percentage points each year, which is more than double the current forecasts on average estimates across LMICs [34]. The practice of using modern contraceptives in Bangladesh is still below par. Due to the COVID-19 pandemic's catastrophic effects on family planning services, it is more important than ever to make bold, new family planning pledges to guarantee that women and girls have access to the high-quality sexual health services they require and deserve. As global health systems focus heavily on protecting and treating people with COVID-19, it is vital that they also protect access to family planning services. A study shows that contraceptive use among reproductive-age women in rural areas of Bangladesh during the COVID-19 era declined by approximately 23% compared to before COVID-19 data [37]. To achieve the goal of 75% of modern contraceptive usage, efforts at all levels have to be reignited.

In our study, we also found heterogeneity due to the high prevalence of contraceptive uses in some studies than other studies. In the case of condom contraceptive methods, 10–49 aged ever-married women were included in Rahman [35]. In this study, it is observed that the age of the women, number of living children, education, religion, media, place of residence, and wealth index have significant impacts on condom contraceptive methods used [17]. Another study was conducted by Koenig et al. [21] in 1992 and at a region in MATLAB. Since this study was conducted in the’90 s and included only one region, the prevalence of contraceptive methods is far distant from other studies and creates heterogeneity. Moreover, a study conducted by Laskar et al. [23] only incorporated employed women and that is the main reason for showing the high prevalence of contraceptive uses like IUDs and male sterilization and withdrawal [23]. From all previous explanations, we observed that heterogeneity is produced due to some factors and sample selection in some cases.

The govt. of Bangladesh and the public health department must develop a comprehensive plan to address the various issues that simultaneously affect the use of contraceptives. Providing contraceptive distribution services through family planning workers, integrating women with non-governmental organizations, and giving priority to rural areas in addition to greater awareness campaigns could all enhance the usage of contraceptives [20]. In addition to mass media campaigns, special content on contraceptive use can be developed and shared on social media platforms to reach relatively young populations.

This study has a few limitations. The main limitation of this study is that, for some of the contraceptive methods, very few studies were found for analysis. Second, a few studies were conducted about 20 years ago. The prevalence estimates of those studies may contribute to a lower overall prevalence of contraceptive usage. However, those studies were required to show the trend in contraceptive use over the years (urban–rural disparities). Third, the analyses (funnel plots) show some publication biases for contraceptive methods.

## Conclusion

This research examined contraceptive usage data for the past three decades in Bangladesh. While the country has made notable strides in increasing overall contraceptive usage, the prevalence and use of modern methods remain low, particularly in urban areas. There is also a significant disparity between urban and rural areas. Observations indicate a lack of enthusiasm for condoms as a short-term contraceptive method. The COVID-19 pandemic has shifted attention away from family planning and reproductive health services. Consequently, there is evidence of a decline in family planning and contraceptive use in Bangladesh. A range of solutions, both supply-side and demand-side, can assist the national family planning program in overcoming these barriers. To achieve the targets of SDGs and family planning-2030 (fp2030), the Bangladesh government must rekindle its commitment to improving reproductive health services and reducing disparities in contraceptive usage across social and geographical lines.

### Supplementary Information

**Additional file 1. **Forest and Funnel plots of different types of contraceptive methods.
