# Supplementary material for: Contraceptive uses among married women in Bangladesh: a systematic review and meta-analyses
Source: J Health Popul Nutr. 2024 Jan 17;43:10. doi: 10.1186/s41043-024-00502-w (PMC10795415; doi:10.1186/s41043-024-00502-w)
Supplement: Supplementary file 1 — Additional file 1. Forest and Funnel plots of different types of contraceptive methods. [file 41043_2024_502_MOESM1_ESM.docx]

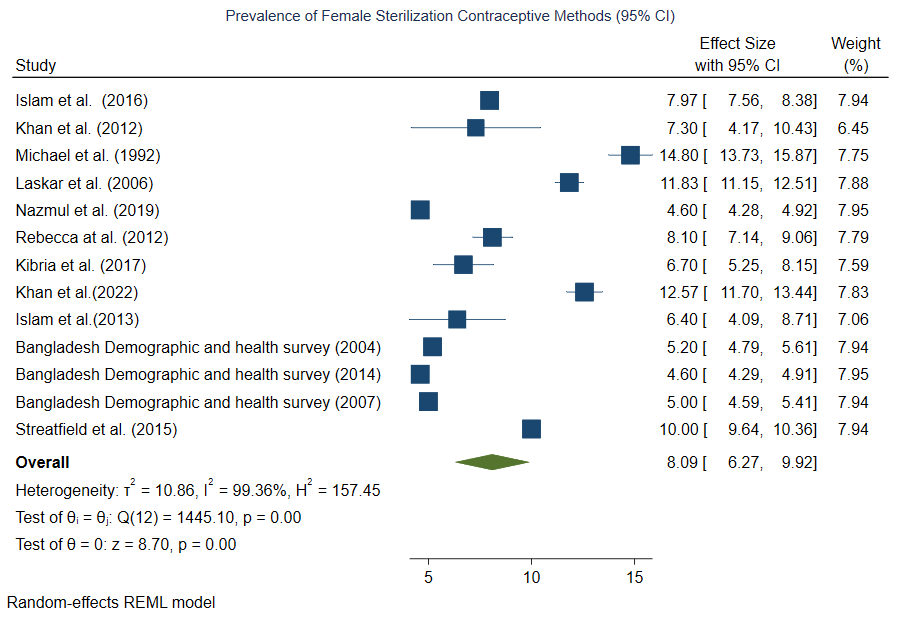


Figure 7: Forest plot showing the results of the pooled prevalence of Female Sterilization methods of contraceptive uses among women in Bangladesh.


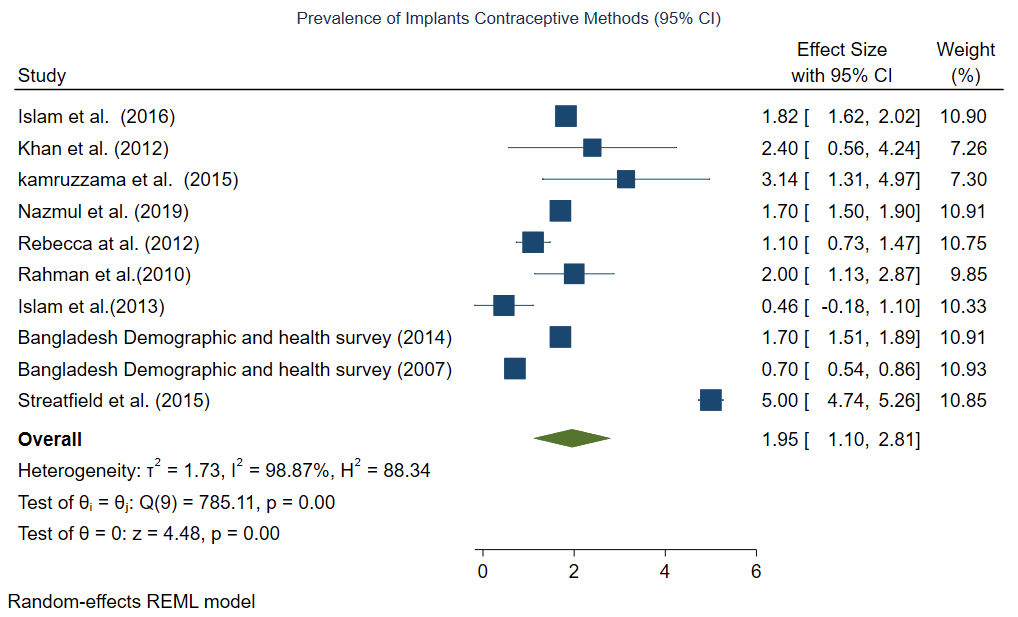


Figure 8: Forest plot showing the results of the pooled prevalence of Implants methods of contraceptive uses among women in Bangladesh.


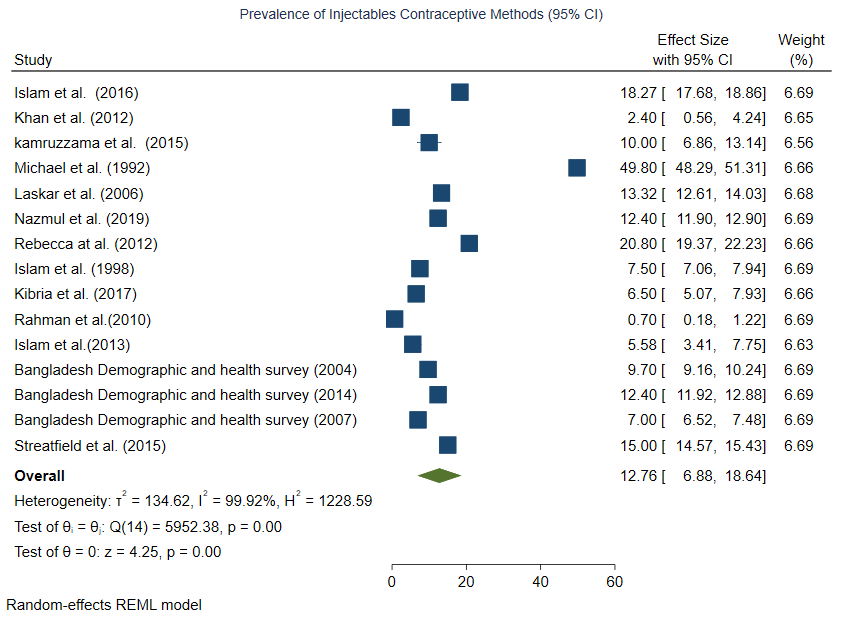


Figure 9: Forest plot showing the results of the pooled prevalence of Injectables methods of contraceptive uses among women in Bangladesh.


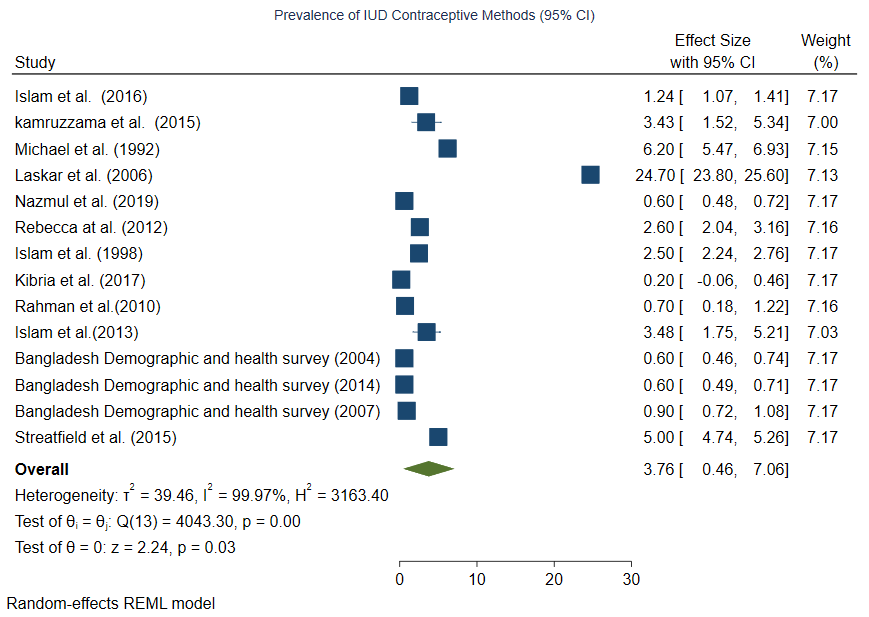


Figure 10: Forest plot showing the results of the pooled prevalence of IUD methods of contraceptive uses among women in Bangladesh.


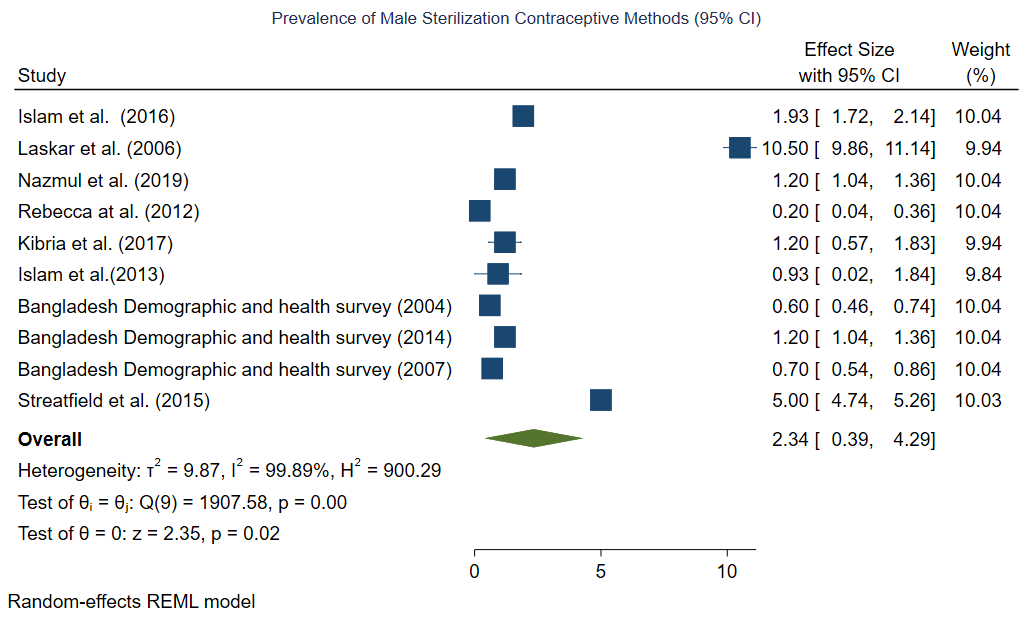


Figure 11: Forest plot showing the results of the pooled prevalence of Male Sterilization methods of contraceptive uses among women in Bangladesh.


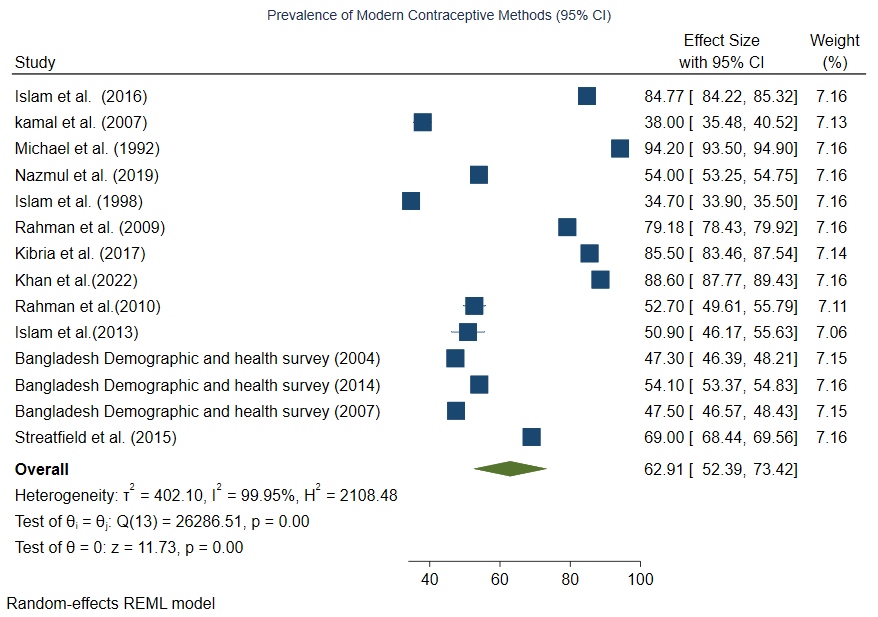


Figure12: Forest plot showing the results of the pooled prevalence of Modern methods of contraceptive uses among women in Bangladesh.


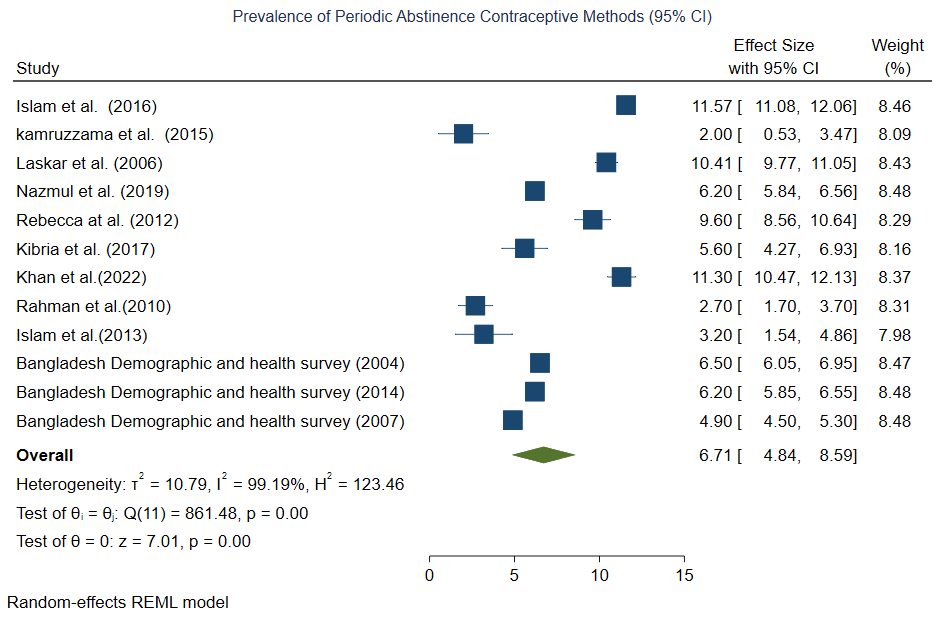


Figure 13: Forest plot showing the results of the pooled prevalence of Periodic Abstinence methods of contraceptive uses among women in Bangladesh.


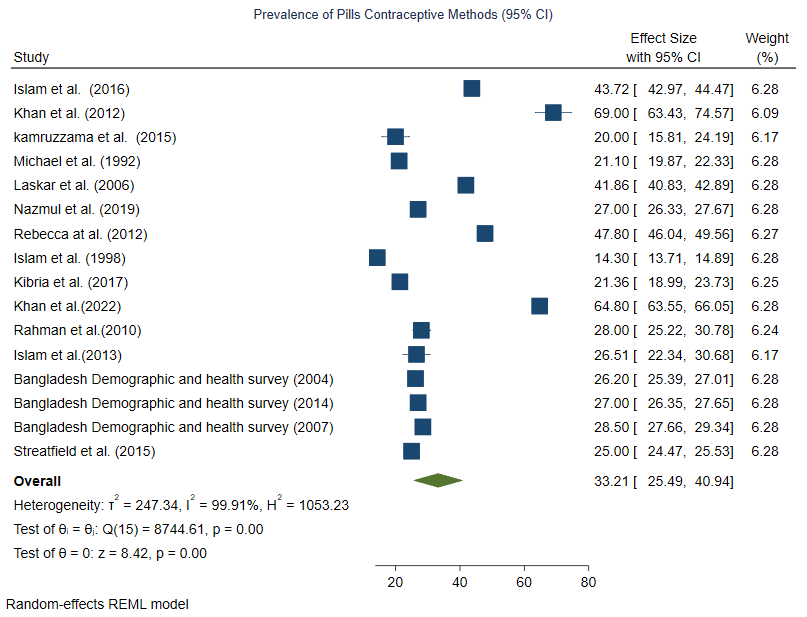


Figure 14: Forest plot showing the results of the pooled prevalence of Pills methods of contraceptive uses among women in Bangladesh.


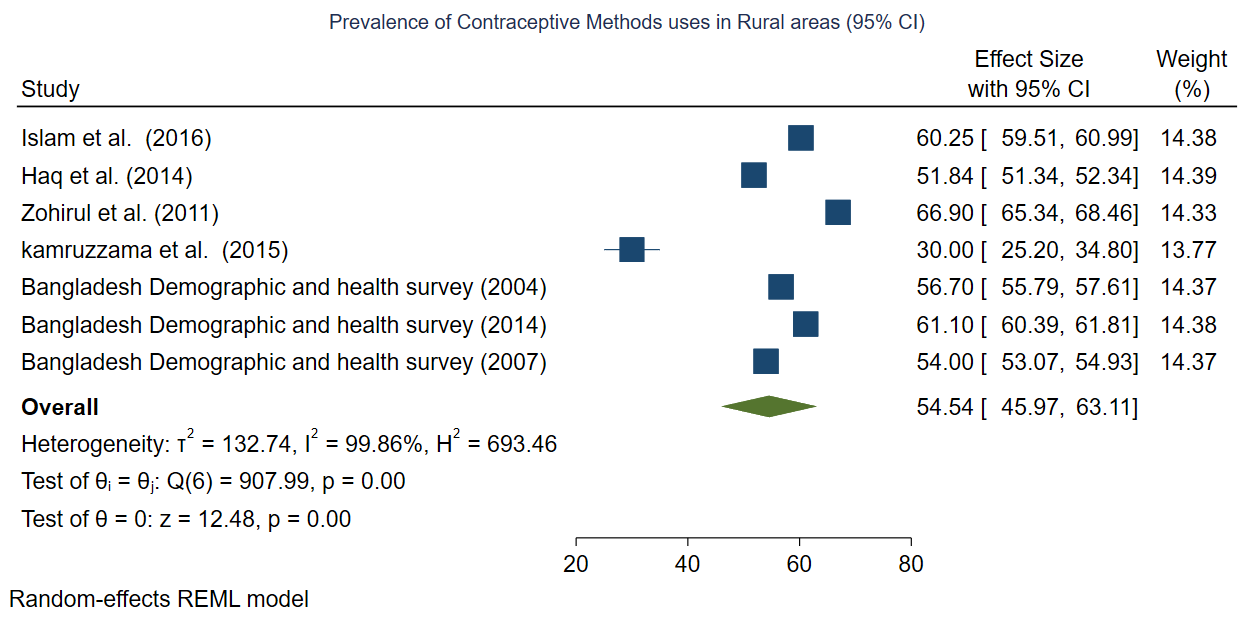


Figure 15: Forest plot showing the results of the pooled prevalence of contraceptive uses in Rural areas among women in Bangladesh.


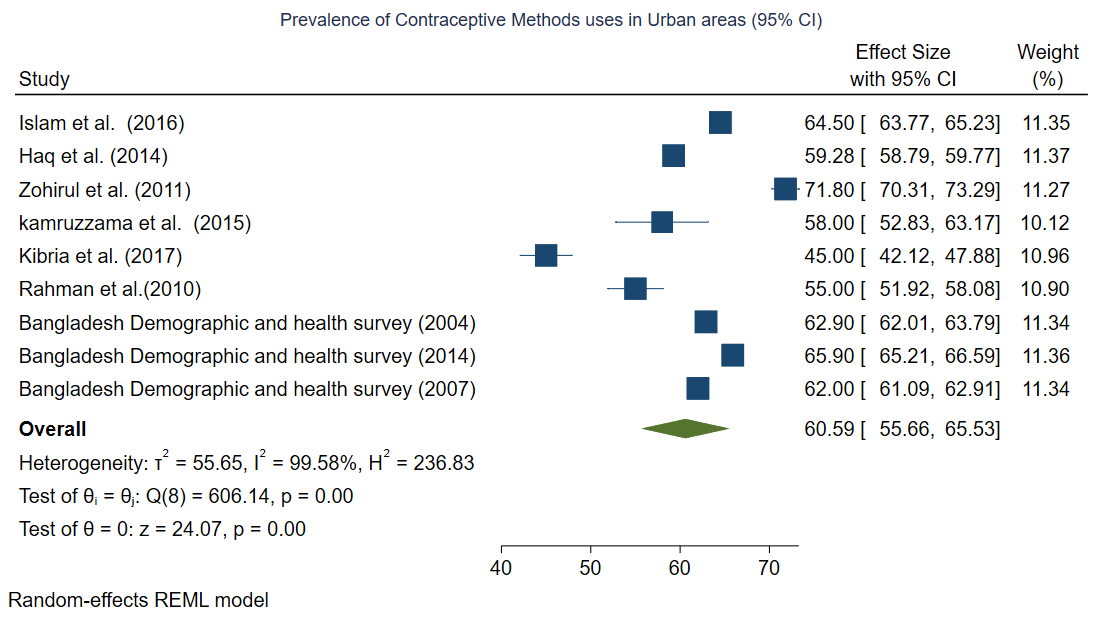


Figure 16: Forest plot showing the results of the pooled prevalence of contraceptive uses in Urban areas among women in Bangladesh.


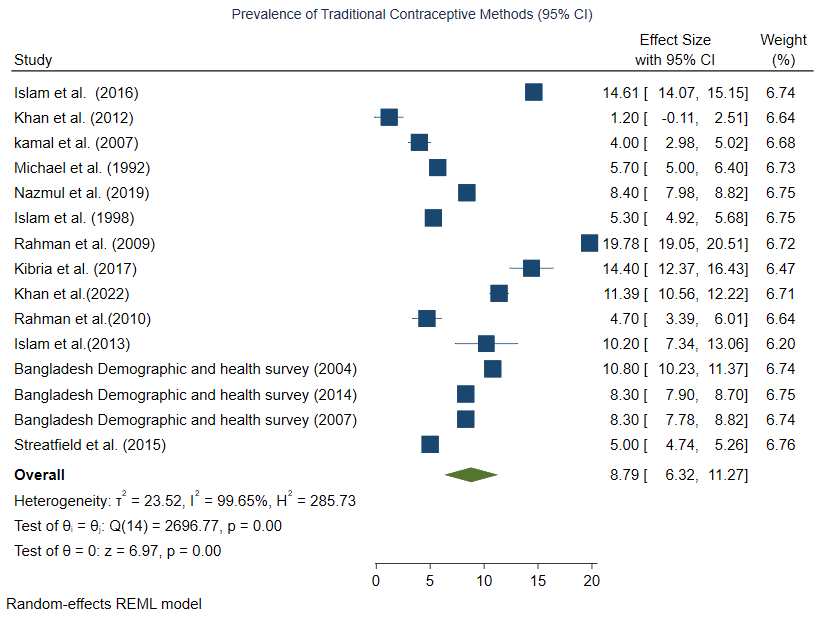


Figure 17: Forest plot showing the results of the pooled prevalence of Traditional contraceptive uses among women in Bangladesh.
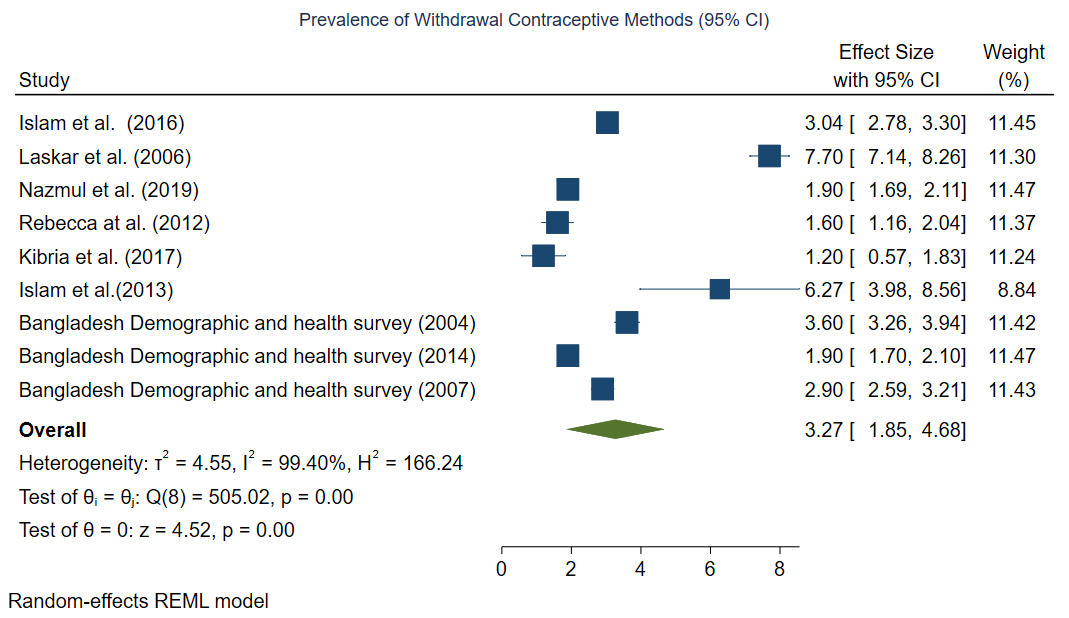
Figure 18: Forest plot showing the results of the pooled prevalence of Withdrawal methods of contraceptive uses among women in Bangladesh.


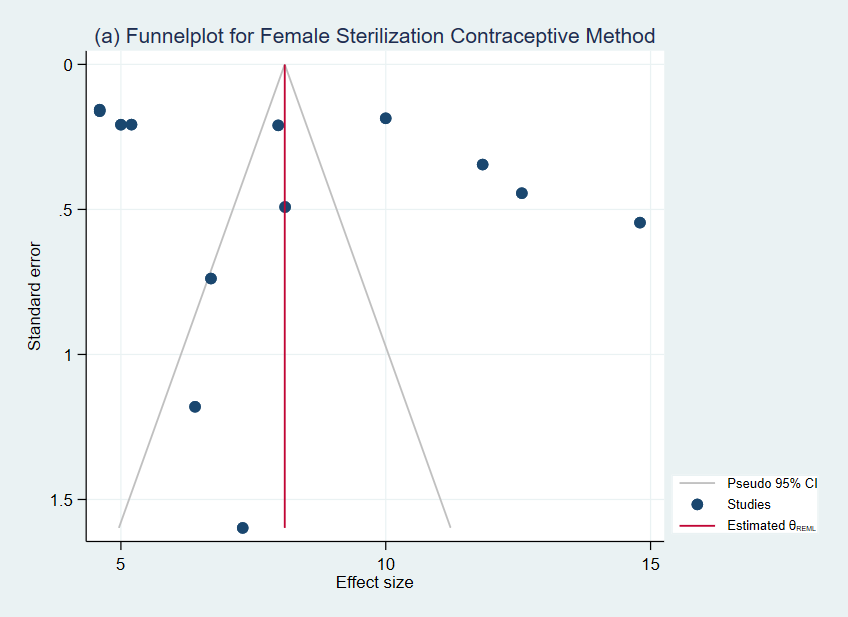


Figure 19: Funnel plot of result of the prevalence of Female Sterilization methods of contraceptive uses among women in Bangladesh.


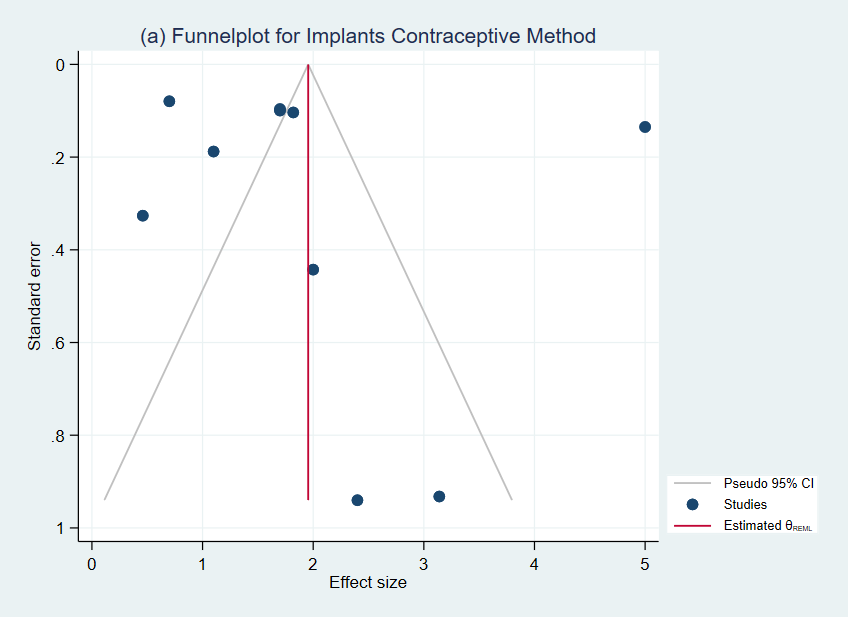


Figure 20: Funnel plot of result of the prevalence of Implants methods of contraceptive uses among women in Bangladesh.


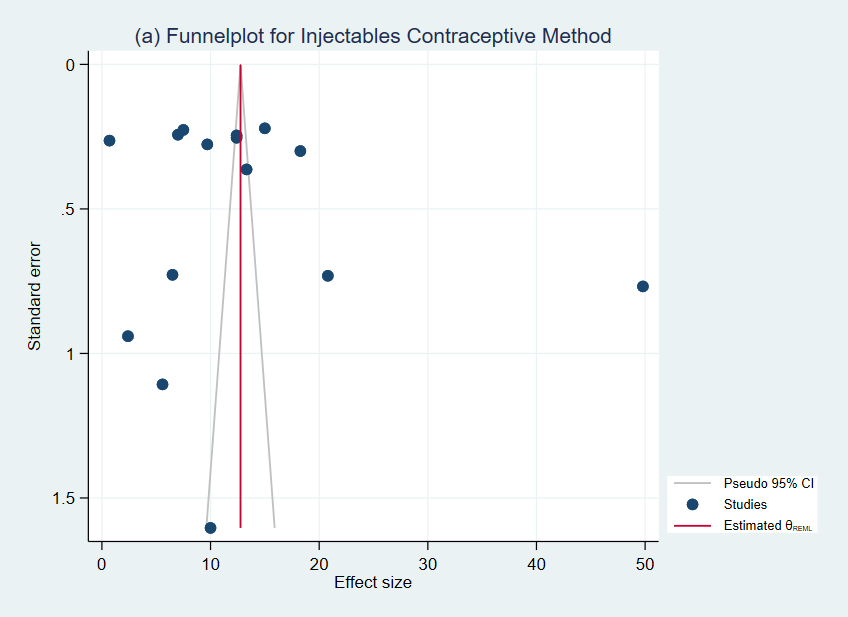


Figure 21: Funnel plot of result of the prevalence of Injectables methods of contraceptive uses among women in Bangladesh.


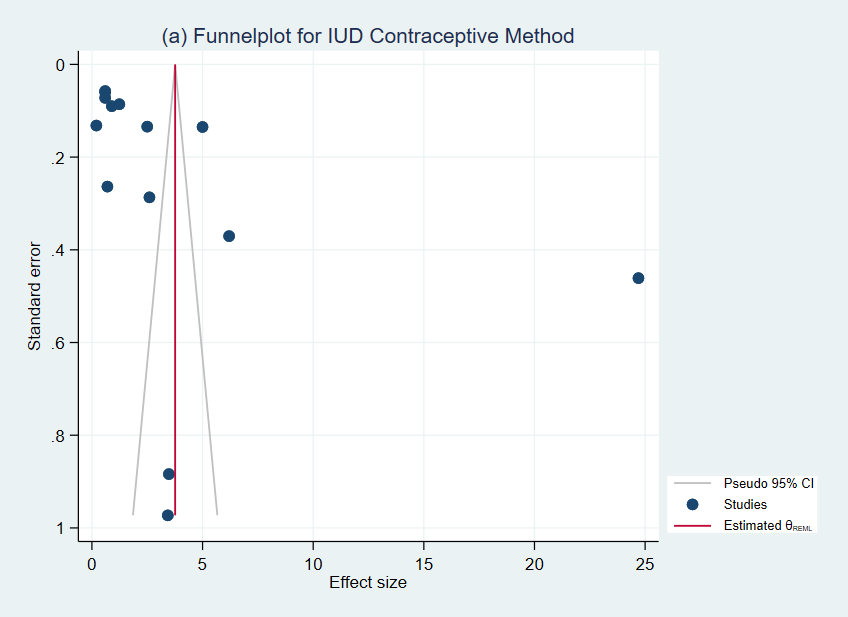


Figure 22: Funnel plot of result of the prevalence of IUD methods of contraceptive uses among women in Bangladesh.


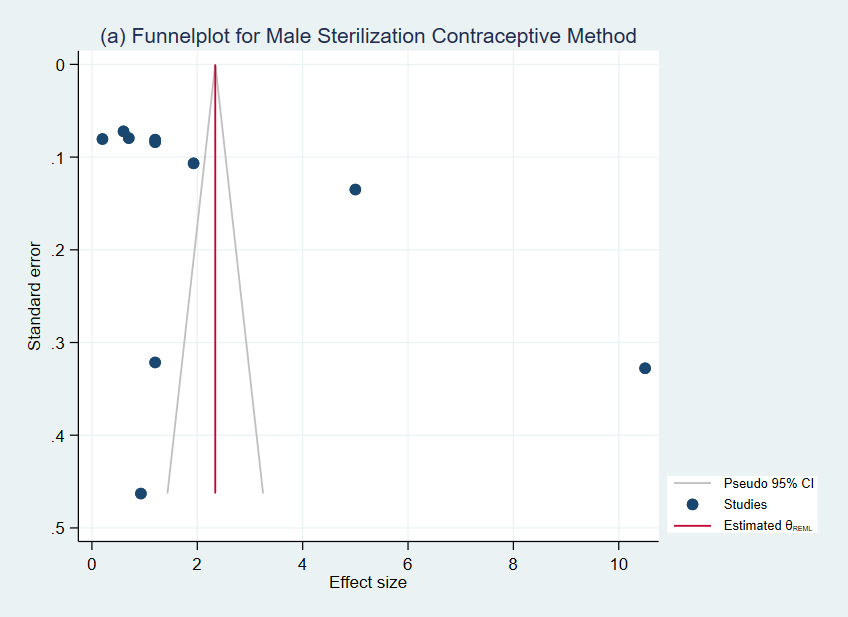


Figure 23: Funnel plot of result of the prevalence of Male Sterilization methods of contraceptive uses among women in Bangladesh.


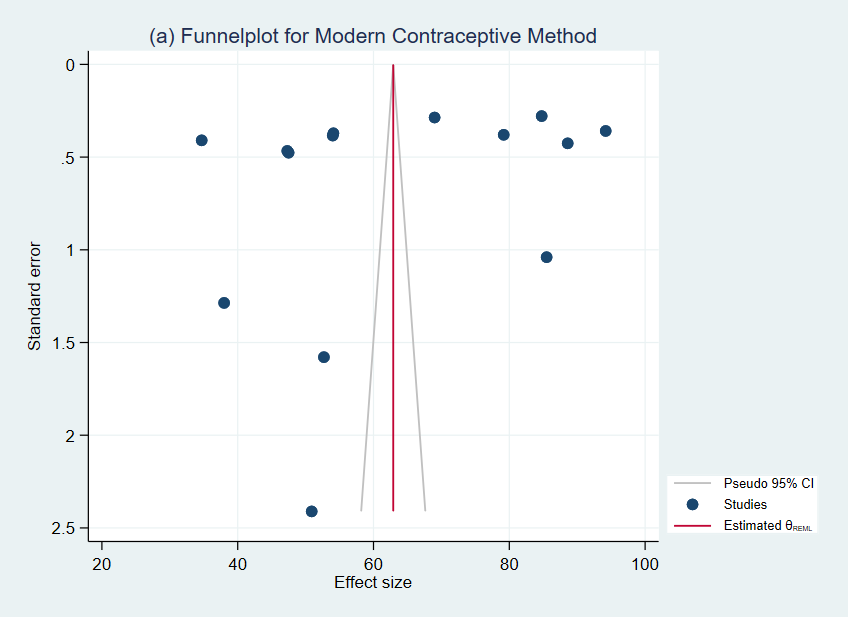


Figure 24: Funnel plot of result of the prevalence of Modern methods of contraceptive uses among women in Bangladesh.


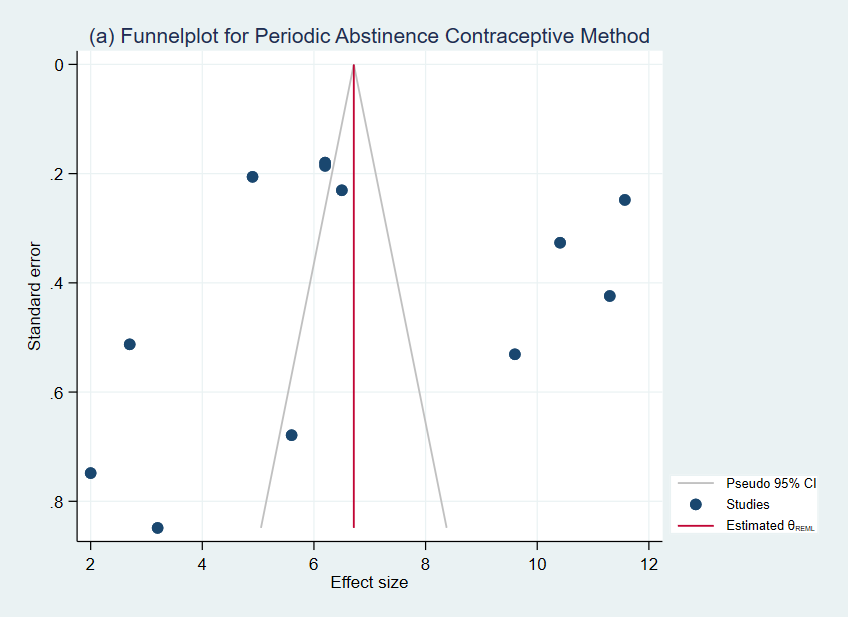


Figure 25: Funnel plot of result of the prevalence of Periodic Abstinence methods of contraceptive uses among women in Bangladesh.


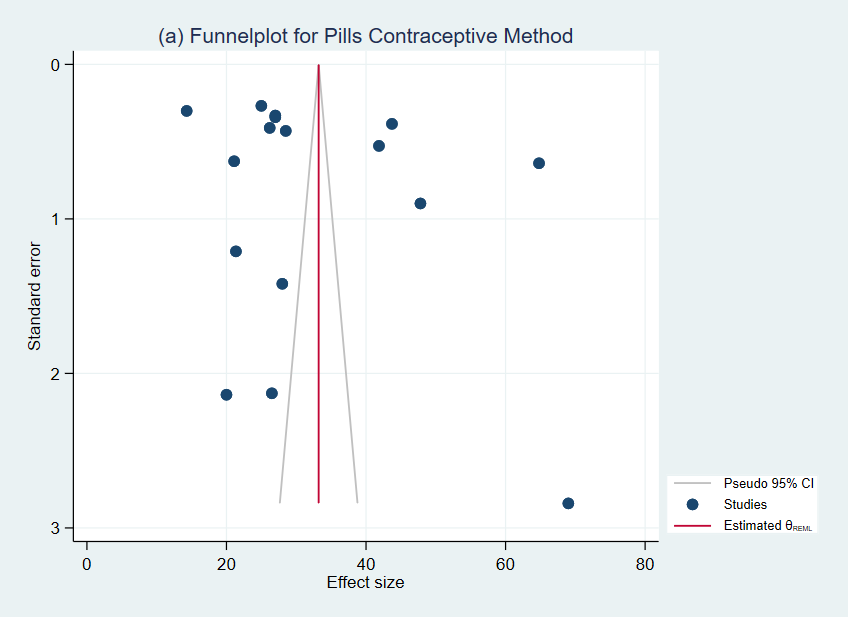


Figure 26: Funnel plot of result of the prevalence of Pills methods of contraceptive uses among women in Bangladesh.


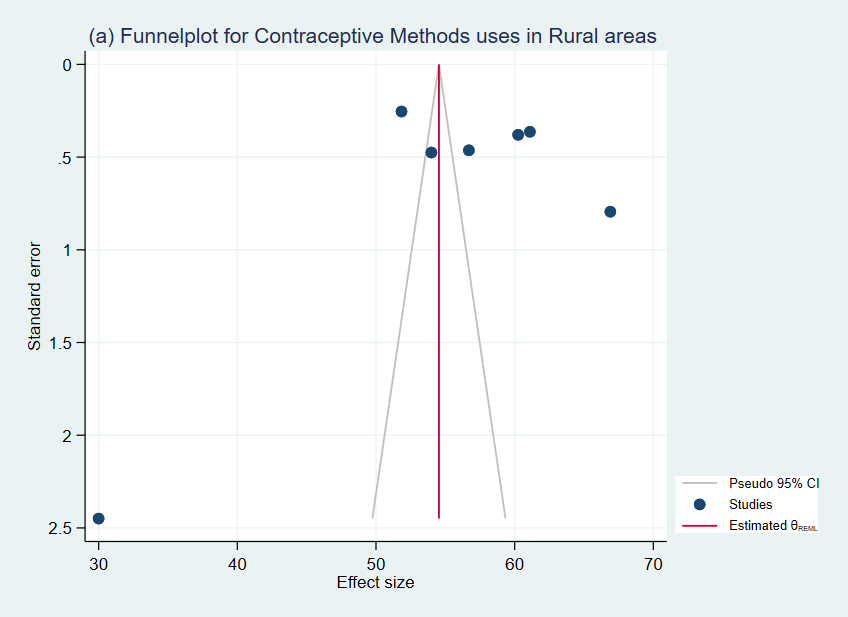


Figure 27: Funnel plot of result of the prevalence of contraceptive uses among Rural areas women in Bangladesh.


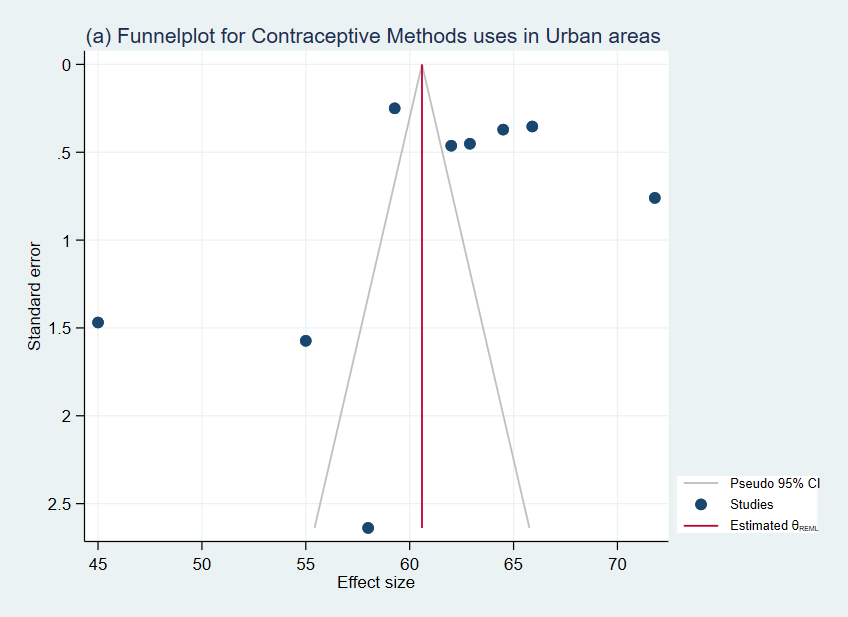


Figure 28: Funnel plot of result of the prevalence of contraceptive uses among Urban areas women in Bangladesh.


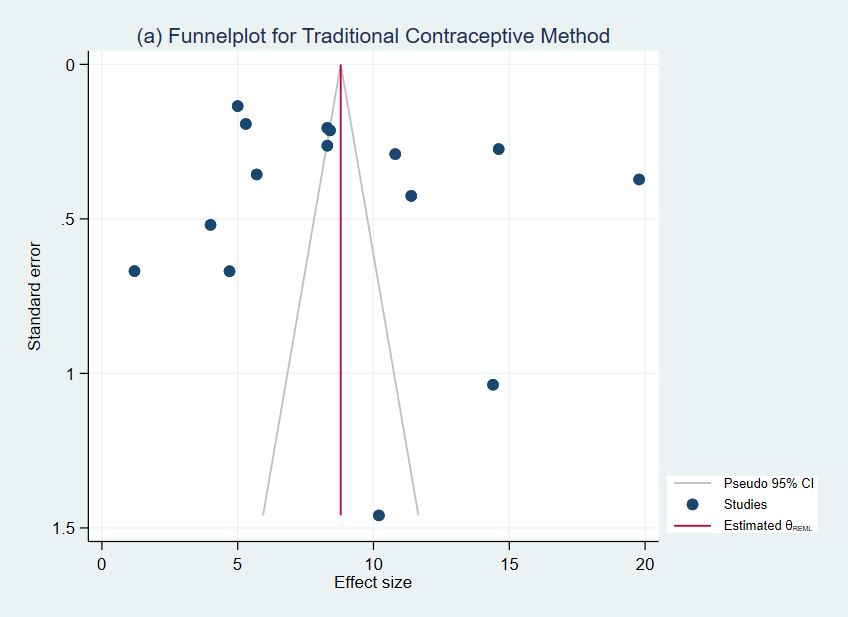


Figure 29: Funnel plot of result of the prevalence of Traditional contraceptive uses among women in Bangladesh.


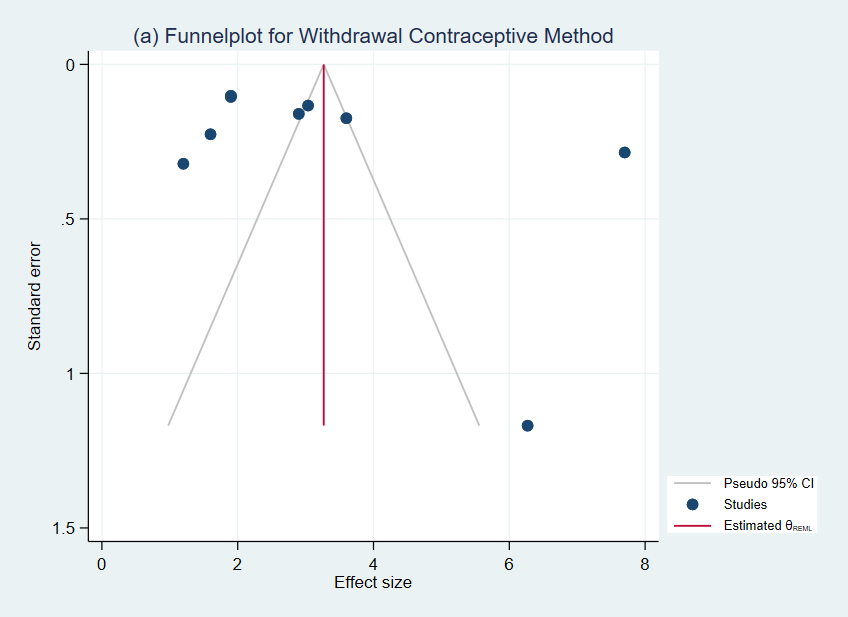


Figure 30: Funnel plot of result of the prevalence of Withdrawal methods of contraceptive uses among women in Bangladesh.
